# Factors Associated with Mortality among Patients on TB Treatment in the Southern Region of Zimbabwe, 2013

**DOI:** 10.1155/2017/6232071

**Published:** 2017-03-02

**Authors:** Kudakwashe C. Takarinda, Charles Sandy, Nyasha Masuka, Patrick Hazangwe, Regis C. Choto, Tsitsi Mutasa-Apollo, Brilliant Nkomo, Edwin Sibanda, Owen Mugurungi, Anthony D. Harries, Nicholas Siziba

**Affiliations:** ^1^AIDS & TB Department, Ministry of Health & Child Care, Harare, Zimbabwe; ^2^International Union against Tuberculosis and Lung Disease, Paris, France; ^3^Ministry of Health and Child Care, Harare, Zimbabwe; ^4^World Health Organisation Country Office, Harare, Zimbabwe; ^5^Health Services Department, Bulawayo, Zimbabwe; ^6^Department of Clinical Research, London School of Hygiene and Tropical Medicine, London, UK

## Abstract

*Background*. In 2013, the tuberculosis (TB) mortality rate was highest in southern Zimbabwe at 16%. We therefore sought to determine factors associated with mortality among registered TB patients in this region.* Methodology*. This was a retrospective record review of registered patients receiving anti-TB treatment in 2013.* Results*. Of 1,971 registered TB patients, 1,653 (84%) were new cases compared with 314 (16%) retreatment cases. There were 1,538 (78%) TB/human immunodeficiency virus (HIV) coinfected patients, of whom 1,399 (91%) were on antiretroviral therapy (ART) with median pre-ART CD4 count of 133 cells/uL (IQR, 46–282). Overall, 428 (22%) TB patients died. Factors associated with increased mortality included being ≥65 years old [adjusted relative risk (ARR) = 2.48 (95% CI 1.35–4.55)], a retreatment TB case [ARR = 1.34 (95% CI, 1.10–1.63)], and being HIV-positive [ARR = 1.87 (95% CI, 1.44–2.42)] whilst ART initiation was protective [ARR = 0.25 (95% CI, 0.22–0.29)]. Cumulative mortality rates were 10%, 14%, and 21% at one, two, and six months, respectively, after starting TB treatment.* Conclusion*. There was high mortality especially in the first two months of anti-TB treatment, with risk factors being recurrent TB and being HIV-infected, despite a high uptake of ART.

## 1. Introduction

Tuberculosis remains one of the world's biggest public health threats and now ranks alongside HIV as the world's leading infectious cause of death. In 2014, there were an estimated 9.6 million people diagnosed with TB globally, of whom 1.5 million died from the disease in the same year (0.4 million were HIV-positive). Despite this, TB incidence has slowly declined by 18% since 2000 and it is estimated that 43 million lives were saved between 2000 and 2014 through effective diagnosis and treatment [1]. However, given that most deaths from TB are preventable, the death toll from the disease is still unacceptably high and efforts to combat this must be continuously accelerated beyond the Millennium Development Goal (MDG) targets, in line with the recently set Sustainable Development Goal (SDG) and the End TB strategy targets [2]. By 2030, the ambition is to reduce TB mortality by 90% compared with 2015. Of the 6 million new cases of TB notified to World Health Organisation (WHO) in 2014, 21% were from the African region which had the highest burden of TB at 281 cases per 100,000 population compared to the global average of 133 cases per 100,000 population [1].

Zimbabwe, which is in the Southern African region, is among the 30 high-burden TB, HIV, and MDR-TB countries in the world and it also has a high HIV prevalence of 15% [3]. In 2014, Zimbabwe had an estimated TB incidence of 278 (193–379) cases per 100,000 population inclusive of HIV-positive TB cases, whilst TB prevalence in the same year was 292 (158–465) cases per 100,000 population [1]. Furthermore, the estimated TB mortality in 2014 stood at 50 (34–68) cases per 100,000 population, although Zimbabwe was notably one of the only 11 high-burden countries to meet the target of halving the TB mortality rate by 2015 compared to 1990. The high burden of TB in Zimbabwe is mostly associated with HIV, with 69% of TB patients having HIV coinfection in 2014 [1]. Thus, national ART guidelines state that all TB/HIV coinfected patients are eligible for ART and cotrimoxazole preventive therapy (CPT) in order to improve TB treatment success rates [4].

The TB treatment success rate for newly registered cases in 2012 was 81% [5], which is below the 85% target recommended by WHO. According to national data, one of the contributors to this low treatment success rate was a high mortality rate of 8% among patients on TB treatment regardless of type and category of TB. Further analysis of this data showed that death rates were highest in the three southern region provinces of Zimbabwe which are Matabeleland North, Matabeleland South, and Bulawayo, each recording case fatalities of 16%, 18%, and 14%, respectively (source: National TB Programme). Death rates in these three provinces have been consistently high in previous years. Given these high mortality rates, we set out to determine among TB patients registered in the three provinces in 2013: (i) their demographic and clinical characteristics, (ii) factors associated with mortality, and (iii) among those who died their time from start of anti-TB treatment to death.

## 2. Methods

### 2.1. Study Design

This was a retrospective cohort study using routinely collected data on patients receiving TB treatment.

### 2.2. Setting: General and Study Site

Selected for this study were three districts from each of the three provinces with the highest TB mortality rates in Zimbabwe in 2013 (nine districts alltogether). Matabeleland South, Matabeleland North, and Bulawayo provinces jointly constitute 16% of Zimbabwe's population according to the Zimbabwe 2012 national census results [6] and have populations of 685,046, 743,871, and 655,675, respectively. Matabeleland South and North provinces are divided into 7 rural districts. Each of these districts has a district hospital and in some instances, there are also mission hospitals at the second level of the health delivery tiered system where there are medical doctors and laboratory facilities enabling the diagnosis of TB through direct smear microscopy and of late GeneXpert MTB/RIF.

The two provinces each have a provincial hospital where referrals from district level are sent. Bulawayo city is Zimbabwe's second largest city where three of the country's central hospitals are located. In addition, there are a number of polyclinics which fall under the Bulawayo City Health Directorate and which are responsible for TB treatment in Bulawayo city. All health facilities in the three provinces offer general health services including TB treatment services as part of an integrated care package.

### 2.3. Management of TB Cases and HIV Coinfection in Zimbabwe

In Zimbabwe, all public health facilities offer TB treatment services integrated with general health services. First, all presumptive pulmonary TB cases at each health centre have their sputum collected, and sputum specimens are sent to the nearest TB diagnosis laboratory for direct smear microscopy or GeneXpert MTB/RIF with results expected to return to their respective health centres within 2 days. Patients diagnosed with TB are then entered in the health facility DOT register and started on TB treatment. Those with smears positive for acid-fast bacilli are diagnosed as smear-positive PTB, whilst those with negative smears or smears not done are referred for a chest radiograph. If the latter is suggestive of TB, the patients may be diagnosed as smear-negative PTB or PTB with smears not done. Extrapulmonary TB (EPTB) is mainly diagnosed on clinical grounds and circumstantial evidence along with supporting specific diagnostic tests [7].

Patients are also classified and recorded by category of TB as follows: (i) new case defined as a patient who has never had treatment for TB or who has taken anti-TB drugs for less than 1 month, or (ii) previously treated case defined as a patient who has received 1 month or more of anti-TB drugs in the past. Patients who are previously treated for TB (named as “retreatment cases”) are further classified by the outcome of their most recent course of treatment either as relapse, treatment after default, treatment after failure, transfer-in, or retreatment others [8].

Upon starting TB treatment, patients take directly observed treatment (DOT) using standardized anti-TB regimens [8], regardless of HIV status. Monitoring is done bacteriologically for all forms of TB since all patients on anti-TB treatment are supposed to have their sputum tested at 2, 4, and 5 months. Smear-negative patients who complete treatment and smear-positive patients who complete treatment with negative smears are regarded as “successfully completing treatment.”

All presumptive TB patients are offered HIV counselling and testing (opt-out provider-initiated) [9] and, if found to have TB/HIV coinfection, CPT is started together with anti-TB treatment, provided there is no contraindication. After starting TB treatment, all HIV coinfected patients should be referred for ART initiation and provision of HIV/AIDS care and support within that health facility or alternatively to the nearest ART initiating clinic within their district if ART services are not available on site.

According to national guidelines, ART should be started at least two weeks after the start of TB therapy that is during the intensive phase when the patient has stabilized on anti-TB treatment regardless of the CD4 cell count status. However, for severely immunosuppressed patients (those with CD4 < 50 cells/uL), ART is initiated within the first two weeks of initiating TB treatment. In 2013, ART initiation services were found at all district, mission, and rural hospitals and selected primary care facilities in the three provinces although as of 2015 all health facilities are now offering ART services.

### 2.4. Study Population

All TB patients who were registered in the district TB registers from the nine selected districts in the three provinces and started on TB treatment between 1 January and 31 December 2013 were included in the study.

### 2.5. Data Variables, Sources of Data, and Data Collection Procedures

Sources of data included TB and ART registers and, in cases of missing data for ART initiation, the ART patient files. Data were abstracted from routinely existing health facility records into structured forms by three teams of trained and experienced data collectors, each consisting of 5 people. First, data were abstracted from district TB registers for each selected district and variables collected from these registers included TB treatment start date, patient age and sex, TB category and type, TB treatment outcome, other comorbidities, HIV status, pre-ART CD4 cell count, WHO stage, and whether the patient was on ART or not. Patient names were also collected and used to follow up and search for missing information about pre-ART CD4 cell count, WHO clinical staging, and ART status. These names were used to search ART registers and individual ART care booklets from the respective health facilities in each district where these patients were registered and receiving anti-TB treatment.

### 2.6. Statistical Analysis

Patient data collected in the structured report form was coded and single-entered by six data entry clerks into EpiData version 3.1 (EpiData, Odense, Denmark) and later cleaned for errors and analysed using STATA, version 13.1 (Stata Corporation, College Station, Texas). Proportions and their 95% confidence intervals (CIs) were reported for categorical variables whilst medians and interquartile ranges were reported for continuous skewed variables. Associations between categorical variables were determined using the chi-square test or alternatively Fisher's Exact test.

Univariate and multivariate-adjusted risk ratios together with their 95% CIs and aimed at determining factors associated with mortality were calculated using generalized linear models with a log link and binomial distribution. If there was a convergence problem with the regression models, STATA's* “binreg”* command was implemented. Variables included in the multivariate generalized linear models were those with a *p* value < 0.25. The multivariate generalized linear model for HIV status, classification, and category of TB included all study participants and were adjusted for age and level of healthcare facility. However, separate multivariate generalized linear models for the variables* ART use recorded, ART initiation in relation to start of TB treatment, WHO staging, and CD4 cell count at ART initiation *whilst adjusting for age, level of healthcare, and TB category were run separately to avoid collinearity and were also restricted to those HIV-positive since they did not apply to those HIV-negative.

For survival analysis, we used date of starting TB treatment as time of entry and follow-up was censored at either date of death or date last seen or date of completing TB treatment. Time-to-event data were complete for 98% of all individuals who successfully completed TB treatment or who died after excluding those who were lost to follow-up, were not evaluated, and were still on MDR-TB treatment. Whilst those excluded should have been included in the survival analysis as censored, this was not possible because their dates of last DOT visit were not recorded in the TB registers. Stratified Kaplan-Meier curves were used to graphically assess survival proportions and calculate the median survival time on TB treatment. A *p* value <0.05 was considered to indicate statistical significance.

### 2.7. Ethical Considerations

Ethics approval was granted locally by the Medical Research Council of Zimbabwe and the International Union against Tuberculosis and Lung Disease (The Union) Ethics Advisory Group, Paris, France. Privacy and confidentiality of information drawn from the hospital registers were ensured by excluding patient names from the electronic data entry. All data abstracted were kept in a safe and secure place accessible only to the investigator.

## 3. Results

There were 1,971 patients who were registered and started on TB treatment between 1 January and 31 December 2013 in the selected districts of the three southern provinces. Demographic and clinical characteristics of TB patients in the southern region provinces are shown in Tables 1 and 2, respectively. Males constituted 1,075 (55%) of all patients whilst the overall median age of patients was 34 years (IQR, 28–44). Close to half of all patients were receiving anti-TB treatment at rural health clinics, whilst 436 (22%) and 341 (17%) were registered at district/provincial hospitals and mission/rural hospitals, respectively. Only 63 (3%) of all patients had a recorded history of travel outside Zimbabwe, and of these 57 (90%) had been to South Africa.

Clinical characteristics of TB patients are shown in Table 2. Of all TB patients, 1,653 (84%) were newly registered TB patients, and of these the majority (44%) were smear-negative pulmonary TB cases whilst 37% were smear-positive PTB cases. Of the retreatment TB cases, 191 (62%) were “retreatment others” whilst 78 (25%) were relapse TB cases. Close to all patients had an HIV test result, of whom 1,538 (78%) were HIV-positive. Of these, 1,399 (91%) were recorded as receiving ART. More than half of all HIV-positive patients had missing WHO clinical staging and baseline CD4 cell counts at ART initiation. Of those with recorded WHO clinical staging, 84% had advanced HIV disease whilst the median CD4 cell count was 133 cells/uL (IQR, 46–282). Of those patients with documented ART status, 584 (42%) were on ART prior to starting TB treatment whilst 543 (39%) initiated ART within 2 weeks of commencing TB treatment.

**Figure 1:**
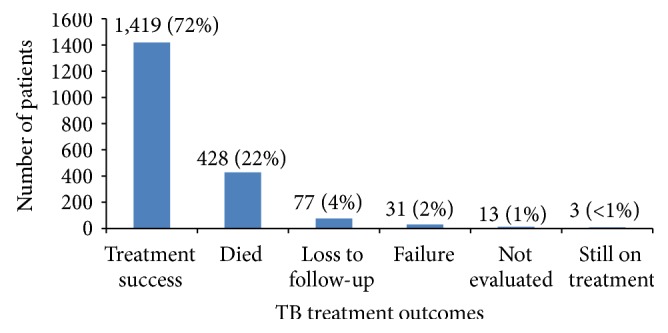
Overall TB treatment outcomes among enrolled patient on TB treatment in southern Zimbabwe, 2013. TB = tuberculosis.

As shown in Figure 1, 1,419 (72%) of all TB patients had a treatment success whilst death (22%) was the most common adverse TB treatment outcome.  Table 3 shows relative risks and their 95% CIs for factors associated with mortality. Patients aged 65 years and older compared to those <5 years old and having retreatment TB compared to newly treated TB were associated with higher risk of mortality in all multivariate regression models. However accessing treatment from a polyclinic or higher level health centre and having CD4 count >50 cells/mL versus ≤50 cells/mL were associated with lower risk of mortality. Being HIV-positive was associated with higher mortality compared to being HIV-negative [adjusted relative risk (ARR) = 1.87 (95% CI, 1.44–2.42)], but among those who were HIV-positive, those on ART had lower mortality [ARR = 0.25 (95% CI, 0.22–0.29)] compared with those not on ART. Compared to those who initiated ART > 3 months prior to starting TB treatment, those who initiated ART within 2 weeks [ARR = 0.73 (95% CI, 0.54–0.98)] were less likely to die whilst those initiated on ART 0–3 months prior to TB treatment were more likely to die [ARR = 1.80 (95% CI, 1.34–2.43)].

**Figure 2:**
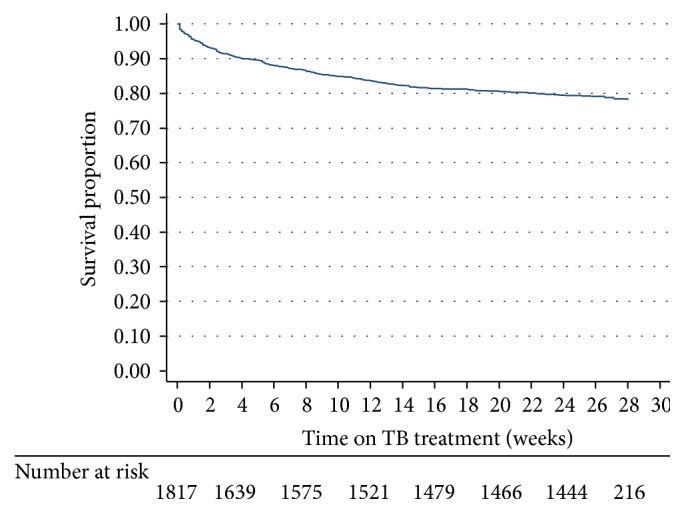
Overall Kaplan-Meier survival estimates among patients on TB treatment. TB = tuberculosis.

**Figure 3:**
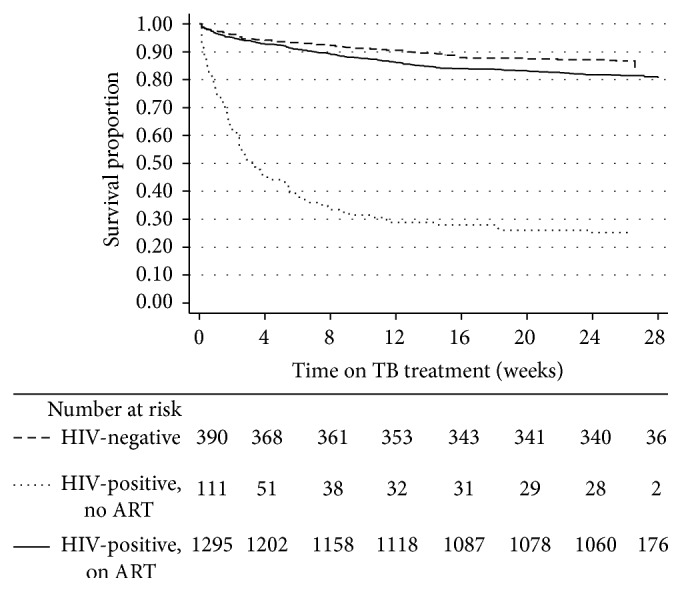
Kaplan-Meier survival estimates stratified by HIV status and ART initiation. TB = tuberculosis; HIV = human immunodeficiency virus; ART = antiretroviral therapy. Note: the numbers of HIV-negative patients and those HIV-positive on ART and not on ART differ from those presented in Table 1 because some patients were excluded from survival analysis because of missing outcome status dates.

The overall mortality incidence rate was 49.1 (44.5–54.2) per 100 person-years.  Figure 2 shows the overall Kaplan-Meier survival estimates for all patients enrolled on TB treatment. The mortality rate increased rapidly in the first two months of TB treatment from 10% at one month to 14% at two months and eventually increased slowly over time to 21% at the 6-month end-point. Kaplan-Meier survival estimates for the HIV-negative compared to those HIV-positive on ART and those HIV-positive but not documented on ART are shown in Figure 3: 10% of the respective cohorts had died by 13 weeks, by 7 weeks, and by less than one week, respectively (*p* < 0.001). In relation to median survival time, this could not be estimated for those HIV-negative and those HIV-positive on ART. However, median survival time among those HIV-positive and not on ART was 3.3 weeks (95% CI, 2.3–5.4).

## 4. Discussion

We conducted a retrospective review of data collected on patients receiving anti-TB treatment in a routine programme setting in the southern region of Zimbabwe where the TB mortality rate is the highest in the country. This region, where the average TB mortality rate is more than double that of the national average of 10% [1], is a cause for concern and attention is needed to address this huge problem. We observed that TB coinfection with HIV among patients on anti-TB treatment was eleven percent higher than the national average in Zimbabwe [5]. This correlates with HIV prevalence figures in this region which are the highest in the country at 21%, 18%, and 19% for Matabeleland South, Matabeleland North, and Bulawayo, respectively, compared to the national HIV prevalence of 15.2% according to the 2010-11 Zimbabwe Demographic and Health Survey [3].

Whilst only a quarter of TB/HIV coinfected patients had a documented CD4 cell count, we observed that these patients presented late with low CD4 cell counts and this is similar to previous local findings [10]. These late presentations occur despite national guidelines recommending over the years an increased CD4 cell count eligibility for starting ART: from a CD4 cell count threshold of <200 cells/mL before 2010 to <350 cells/mL from 2011 to 2013 and eventually to <500 cells/mL from 2014 onwards in accordance with WHO guidelines [11–13]. We also observed that the elderly, those with recurrent TB, those diagnosed HIV-positive, those HIV-positive with no documentation of ART, and those HIV-positive presenting with lower CD4 cell counts were more likely to die during anti-TB treatment. Of particular note was the fact that mortality was most common in the intensive phase of anti-TB treatment particularly for those who were HIV-positive without documented ART initiation.

A major strength of this study is that data were an exhaustive sample of all registered cases on anti-TB treatment in the high mortality burden districts of the three highest TB death rate provinces, and hence this study was well powered. Data were obtained from routinely existing programme data which make the study representative of TB patients receiving treatment in public health facilities in southern Zimbabwe. Study limitations include access to few variables that may not exhaustively provide information on mortality-associated factors and also possibilities of incomplete recording of important patient information such as whether an HIV-positive patient was on ART and when they initiated ART, dates of death, and the presence or absence of other comorbidities (such as diabetes mellitus) which may account for mortality among patients who received anti-TB treatment. Another study limitation was that there were no postmortem facilities or access to sophisticated laboratory diagnostic facilities and therefore the exact cause(s) of death could not be determined.

Being HIV-positive was the most common risk factor for mortality whilst on anti-TB treatment and this has been reported in studies from different parts of the world such as in Cameroon [14], the United States [15], South Africa [16], and Ghana [17]. HIV-positive individuals with latent TB are known to be approximately 20–30 times more likely to develop TB disease than those who are HIV negative, at a rate of 8–10% per year [1], because of their suppressed immune system which can result in an increase odds of mortality with further progression of the HIV disease killing 1 in 3 patients [18]. Starting TB treatment with a low CD4 cell count, particularly those with CD4 cell counts <50 cells/mL, was associated with higher mortality and this is a well-established risk factor for early mortality [19]. The declining CD4 cell count influences both the frequency and severity of active TB disease [20], and active TB disease may be associated with a higher HIV viral load and more rapid progression of HIV disease [21]. Such cases presenting for TB treatment with advanced HIV infection are also likely to present with other AIDS-defining diseases which further add to the risk of dying [22].

In this study we observed a reduction in mortality among HIV-positive TB patients who had documented ART use and this clearly highlights the protective effects of ART from mortality. Similar findings by Agbor et al. [14] showed that those who were HIV coinfected and not receiving antiretroviral therapy were more than twofold likely to die. Among those HIV-infected, starting ART in the intensive phase of TB treatment was also associated with lower mortality compared to those who were previously on ART. These findings concur with SAPiT [23], CAMELIA [24], and STRIDE [25] clinical trials which informed WHO guidance and collectively demonstrated that early initiation of ART can reduce mortality and AIDS progression, notwithstanding the risk of increased immune reconstitution inflammatory syndrome (IRIS) in patients with active TB and with very low CD4 cell counts (i.e., <50 cells/mL). Those who had started TB treatment within 3 months of ART initiation had an almost twofold higher risk of mortality during anti-TB treatment and this may be attributed to late presentation for ART initiation with advanced HIV disease which is associated with “unmasking” TB-IRIS [26] and early mortality within the first three months of ART initiation [19]. It is also possible that there are delays in laboratory diagnosis and start of TB treatment among presumptive TB patients identified through intensified TB-case finding using the WHO recommended four-symptom TB checklist within ART clinics and this requires further investigation in future studies.

Late presentation with advanced HIV disease and low CD4 cell counts at the time of starting anti-TB treatment are often anecdotally reported by health workers in these southern regions of Zimbabwe as the major reason for the high mortality rates. Many of these patients are said to reside as migrant workers in neighbouring South Africa where they do not seek HIV testing and HIV treatment and care services and commonly return to their homes to seek treatment when they are very sick and bed-ridden. Whilst this records review study also sought to determine the proportion of patients who had a history of travel to South Africa as a proxy indicator of these HIV late presentation cases, less than five percent had this information documented and we also did not find it to be a significant risk factor for mortality. It is likely that this information may have been underreported since TB and HIV data collection tools are not standardized to collect this data.

Whilst male gender has commonly been reported as a risk factor for mortality among TB patients in other studies [15, 16, 27], this was not the case in our study. As with other studies, in Sub-Saharan Africa, there was higher mortality among those with pulmonary TB for which sputum smear status was unknown [17] and smear-negative pulmonary PTB [16]. In addition to most smear-negative PTB cases being HIV-positive, there are potential time delays in diagnosis, after receipt of negative sputum smears, which include undergoing a chest radiograph (CXR) that is performed, read, and interpreted by a clinician and a clinical assessment. These diagnosis delays [28] can be associated with progression of disease and worse treatment outcomes. Smear-not-performed PTB cases did not follow national guidelines which recommend TB diagnosis with two sputum smear examinations, and this may result in overdiagnosis of TB disease and underdiagnosis of other respiratory diseases thus increasing mortality risk.

Mortality was also notably higher among patients with recurrent TB [16, 27, 29] who have a strong association with HIV infection, whether on ART or with unknown ART status [30]. Such patients are also more likely to be noncompliant to TB treatment and hence linked with drug-resistant TB [31]. Multidrug-resistant (MDR-TB) poses a threat to TB control worldwide, and globally an estimated 3.5% (95% CI: 2.2–4.7%) of new cases and 20.5% (95% CI: 13.6–27.5%) of previously treated cases have MDR-TB. In 2013, neighbouring South Africa was one of the countries with the highest number of notified MDR-TB or Rifampicin-resistant TB (RR-TB) cases who were eligible for MDR-TB treatment. Only 41% of those diagnosed were enrolled to treatment [5], and this is of great concern given that Zimbabwe had the largest number of registered immigrants in South Africa from the Africa region in 2013 [32]. The incidence of MDR-TB is also increasing in high human immunodeficiency virus (HIV) prevalence settings, with high associated mortality. A recent meta-analysis of treatment outcomes for HIV and MDR-TB coinfected adults and children showed that treatment success rates were low, mortality was 38% in adults (95% CI 28–48.1) and 11.4% (95% CI 5.8–17.1) in children, and loss to follow-up was also higher among adults (16.1%, 95% CI 9–23.2) than among children (3.9%, 95% CI 0.9–6.9) [33]. In our setting, it is possible that mortality among TB patients may be partly driven by undiagnosed drug-resistant TB and hence it is important to scale up the use of Xpert MTB/RIF assays for TB diagnosis especially in HIV settings.

Mortality was also notably higher in those aged >65 years and this could possibly be attributed to diabetes mellitus which is common among the elderly and causes immunosuppression thus increasing the risk of TB disease [34]. The prevalence of diabetes mellitus (DM) in adults for Zimbabwe is unexplored and therefore requires investigation. The association between diabetes mellitus and TB has been well documented, and some studies report that smear-negative PTB is more common in people with diabetes, although this association may require rigorous studies to prove an association [35]. This may pave the way for interventions such as active case finding and treatment of latent TB and efforts to diagnose, detect, and treat DM may have a beneficial impact on TB control. It is also possible that rifampicin-, isoniazid-, and pyrazinamide-containing regimens led to anti-TB drug-induced liver disease in these elderly patients [36], and this in turn can lead to drug-induced acute liver failure which is associated with high mortality [37]. This also will require further exploration in future studies.

There are a number of programmatic implications from this study. First it is important to continue doing HIV testing for all presumptive TB patients given the high HIV coinfection in these settings and subsequently ensuring that all TB patients who are HIV-infected are started on ART as soon as possible after anti-TB treatment. Second, with most clinical management and initiation on ART and TB treatment being done by nurses, it is important to ensure that there is continued clinical mentorship and training of these cadres to manage any arising drug interactions in the treatment of HIV-associated TB or TB-related IRIS given the complexities of patients presenting for TB treatment with advanced HIV disease. Third, it is necessary to scale up the use of the Xpert MTB/RIF assay technology to diagnose TB among HIV-infected patients in order to identify drug-resistant TB so that these patients receive appropriate treatment. This will reduce reliance on the use of sputum smear microscopy which is also less sensitive among HIV-infected TB patients with low CD4 cell counts. Finally, it may be worthwhile exploring the burden of DM among TB patients, particularly among the elderly who are at higher risk of noncommunicable diseases, in order to enable appropriate management.

## Figures and Tables

**Table 1:** Demographic characteristics of TB patients in southern Zimbabwe, 2013.

Characteristics	*n*	*N*	% (95% CI)
Sex			
Female	891	1,966	45.3 (43.1–47.5)
Male	1,075	1,966	54.7 (52.5–56.9)
Missing	5	1,971	0.3
Age (in years)			
0–4	67	1,967	3.4 (2.7–4.3)
5–14	70	1,967	3.6 (2.8–4.5)
15–44	1,353	1,967	68.8 (66.7–70.8)
45–64	366	1,967	18.6 (16.9–20.4)
65+	111	1,967	5.6 (4.7–6.8)
Missing	4	1,971	0.2
*Median (IQR) *	*34 (28–44)*		
Type of health facility			
Rural health clinic	922	1,965	46.9 (44.7–49.1)
Mission/rural hospital	341	1,965	17.4 (15.7–19.1)
Polyclinic	266	1,965	13.5 (12.1–15.1)
District/provincial hospital	436	1,965	22.2 (20.4–24.1)
Missing	6	1,971	0.3
Recorded history of travel outside Zimbabwe prior to TB treatment			
Yes	63	1,971	3.2 (2.5–4.1)
No	1,908	1,971	96.8 (95.9–97.5)

IQR = interquartile range; TB = tuberculosis.

**Table 2:** Clinical characteristics of TB patients in southern Zimbabwe, 2013.

Characteristics	*n*	*N*	Percentage (95% CI)
TB category			
New	1,653	1,969	84.0 (82.3–85.5)
Retreatment	314	1,969	15.9 (14.4–17.6)
MDR-TB	2	1,969	0.1 (0.0–0.4)
Missing data	2	1,971	0.1
Type of new TB			
Smear-positive PTB	603	1,651	36.5 (34.2–38.9)
Smear-negative PTB	720	1,651	43.6 (41.2–46.0)
EPTB	227	1,651	13.7 (12.2–15.5)
Smear-not-performed PTB	101	1,651	6.1 (5.1–7.4)
Missing	2	1,653	0.12
Type of retreatment TB			
Treatment after default	29	308	9.4 (6.6–13.2)
Retreatment others	191	308	62.0 (56.4–67.3)
Relapse	78	308	25.3 (20.8–30.5)
Treatment after failure	10	308	3.2 (1.7–5.9)
Missing data	6	314	1.9
HIV status			
HIV-negative	419	1,971	21.3 (19.5–23.1)
HIV-positive	1,538	1,971	78.0 (76.1–79.8)
Unknown	14	1,971	0.7 (0.4–1.2)
WHO clinical staging at ART initiation^*∗*^			
I	30	638	4.7 (3.3–6.7)
II	75	638	11.8 (9.5–14.5)
III	494	638	77.4 (74.0–80.5)
IV	39	638	6.1 (4.5–8.3)
Missing data	900	1,538	58.5
CD4 count (cells/mL) at ART initiation^*∗*^			
≤50	96	364	26.4 (22.1–31.2)
51–200	137	364	37.6 (32.8–42.8)
201–350	74	364	20.3 (16.5–24.8)
351–500	28	364	7.7 (5.4–10.9)
>500	29	364	8.0 (5.6–11.2)
Missing data	1,174	1,538	76.3
*Median (IQR)*	*132.5 (46; 282)*		
ART use recorded^*∗*^			
No	139	1,538	9.0 (7.7–10.6)
Yes	1,399	1,538	91.0 (89.4–92.3)
ART initiation in relation to start of TB treatment^*∗*^			
>3 months before TB treatment	412	1,399	29.4 (27.1–31.9)
0–3 months before TB treatment	172	1,399	12.3 (10.7–14.1)
<2 weeks after TB treatment	543	1,399	38.8 (36.3–41.4)
2–8 weeks after TB treatment	15	1,399	1.1 (0.6–1.8)
Not recorded	257	1,399	18.4 (16.4–20.5)

CI = confidence interval; TB = tuberculosis; MDR-TB = multidrug resistant TB; PTB = pulmonary TB; EPTB = extrapulmonary PTB; HIV = human immunodeficiency virus; WHO = World Health Organisation; IQR = interquartile range; ART = antiretroviral therapy.

^*∗*^These variables refer only to those who were diagnosed as HIV-positive.

**Table 3:** Factors associated with mortality among TB patients in southern Zimbabwe, 2013.

Characteristics	*N*	Died, *n* (%)	Univariate RR (95% CI)	Multivariate-adjusted RR (95% CI)^*ǂ*^			
				Model 1	Model 2	Model 3	Model 4
Total	1,847	428 (23.2)	—	—	—	—	—
*Age (in years)*							
<5	63	11 (17.5)	Reference	Reference	Reference	Reference	Reference
5–14	69	11 (15.9)	0.91 (0.43–1.96)	0.91 (0.42–1.99)	0.89 (0.43–2.00)	1.76 (0.66–4.66)	1.07 (0.39–2.94)
15–44	1,257	290 (23.1)	1.32 (0.77–2.28)	1.17 (0.65–2.10)	1.16 (0.64–2.09)	2.17 (0.97–4.84)	1.12 (0.47–2.65)
45–64	347	74 (21.3)	1.22 (0.69–2.17)	1.10 (0.60–2.01)	1.12 (0.61–2.4)	2.02 (0.91–4.51)	0.95 (0.39–2.32)
65+	107	41 (38.3)	**2.19 (1.22**–**3.95)**	**2.48 (1.35–4.55)**	**2.47 (1.35–4.57)**	**3.13 (1.4–6.98)**	1.37 (0.5–3.8)
Missing data	4	1 (25)	—	—	—	—	—
*Type of health facility*	1847						
Rural health clinic	870	214 (24.6)	Reference	Reference	Reference	Reference	Reference
Mission/rural hospital	319	79 (24.8)	1.01 (0.80–1.26)	0.96 (0.77–1.20)	1.04 (0.62–1.76)	**0.94 (0.94-0.94)**	0.91 (0.68–1.21)
Polyclinic	254	35 (13.8)	**0.56 (0.40–0.78)**	**0.56 (0.40–0.78)**	**0.22 (0.07–0.7)**	**0.55 (0.39–0.77)**	0.57 (0.39–0.85)
District/provincial hospital	398	98 (24.6)	1.00 (0.81–1.23)	0.99 (0.81–1.21)	**0.33 (0.12–0.9)**	**0.9 (0.9-0.9)**	0.78 (0.59–1.02)
Missing data	6	2 (33.3)	—	—	—	—	—
*TB category*							
New TB	1557	337 (21.6)	Reference	Reference	Reference	Reference	Reference
Retreatment TB	288	90 (31.3)	**1.44 (1.19–1.76)**	**1.34 (1.10–1.63)**	1.2 (0.65–2.22)	**1.53 (1.39–1.68)**	**1.59 (1.24–2.04)**
Missing data	2	1 (50)	—	—	—	—	—
*HIV status*							
HIV-negative	396	59 (14.9)	Reference	Reference	Reference	Reference	Reference
HIV-positive	1439	364 (25.3)	**1.70 (1.32–2.18)**	**1.87 (1.44–2.42)**	—	—	
HIV unknown	1	1 (100)	—	—	—	—	—
Missing data	11	4 (36.4)	—	—	—	—	—
*WHO clinical staging at ARTinitiation* ^*∗*^							
I	27	3 (11.1)	Reference	Reference	Reference	Reference	Reference
II	70	9 (12.9)	1.16 (0.34–3.95)	—	0.65 (0.17–2.43)	—	
III	470	69 (14.7)	1.32 (0.44–3.93)	—	1.22 (0.45–3.31)	—	
IV	39	5 (12.8)	1.15 (0.30–4.43)	—	1.29 (0.36–4.58)	—	
Missing data	831	278 (33.4)	—	—	—	—	—
*CD4 count at ART initiation(cells/mL)* ^*∗*^							
≤50	94	28 (29.8)	Reference	Reference	Reference	Reference	Reference
51–200	132	15 (11.4)	**0.38 (0.22–0.67)**	—	**0.40 (0.23–0.7)**	—	
201–350	68	9 (13.2)	**0.44 (0.22–0.88)**	—	**0.47 (0.24–0.91)**	—	
351–500	27	1 (3.7)	**0.12 (0.02–0.87)**	—	**0.13 (0.02–0.88)**	—	
>500	29	5 (17.2)	0.58 (0.25–1.36)	—	0.61 (0.26–1.46)	—	
Missing data	1,089	306 (28.1)	—	—	—	—	—
*ART use recorded* ^*∗*^							
No	117	90 (76.9)	Reference	Reference	Reference	Reference	Reference
Yes	1,322	274 (20.7)	**0.27 (0.23–0.31)**	—	—	**0.25 (0.22–0.29)**	
*ART initiation in relation to start of TB treatment* ^*∗*^							
>3 months before TB treatment	392	83 (21.2)	Reference	Reference	Reference	Reference	Reference
0–3 months before TB treatment	165	56 (33.9)	**0.84 (0.63–1.12)**	—	—	—	**1.8 (1.34–2.43)**
<2 weeks after TB treatment	515	73 (14.2)	**1.34 (0.99–1.82)**	—	—	—	**0.73 (0.54–0.98)**
2–8 weeks after TB treatment	13	2 (15.4)	**0.56 (0.41–0.76)**	—	—	—	0.88 (0.24–3.19)
Not recorded	237	60 (25.3)	—	—	—	—	—

RR = relative risk; CI = confidence interval; TB = tuberculosis; PTB = pulmonary TB; EPTB = extrapulmonary PTB; HIV = human immunodeficiency virus; WHO = World Health Organisation; ART = antiretroviral therapy.

^*∗*^These variables refer only to those who were diagnosed as HIV-positive.

^*ǂ*^Multivariate generalized linear regression models 2, 3, and 4 exclude HIV-negative and HIV unknown patients.

Model 1 assesses HIV status whilst adjusting for the potential confounding effects of age, type of health facility, and TB category.

Model 2 assesses WHO clinical staging and CD4 count at ART initiation whilst adjusting for the potential confounding effect of age, type of health facility, and TB category.

Model 3 assesses ART use (yes/no) whilst adjusting for the potential confounding effect of age, type of health facility, and TB category.

Model 4 assesses “ART timing in relation to start of TB treatment” whilst adjusting for potential confounding effect of age, type of health facility, and TB category.
